# Extracellular CIRP promotes Kupffer cell inflammatory polarization in sepsis

**DOI:** 10.3389/fimmu.2024.1411930

**Published:** 2024-05-30

**Authors:** Junji Shimizu, Atsushi Murao, Yongchan Lee, Monowar Aziz, Ping Wang

**Affiliations:** ^1^Center for Immunology and Inflammation, The Feinstein Institutes for Medical Research, Manhasset, NY, United States; ^2^Departments of Surgery and Molecular Medicine, Zucker School of Medicine at Hofstra/Northwell, Manhasset, NY, United States

**Keywords:** eCIRP, Kupffer cell, sepsis, acute liver injury, M1

## Abstract

**Introduction:**

Sepsis is a life-threatening inflammatory condition caused by dysregulated host responses to infection. Extracellular cold-inducible RNA-binding protein (eCIRP) is a recently discovered damage-associated molecular pattern that causes inflammation and organ injury in sepsis. Kupffer cells can be activated and polarized to the inflammatory M1 phenotype, contributing to tissue damage by producing proinflammatory mediators. We hypothesized that eCIRP promotes Kupffer cell M1 polarization in sepsis.

**Methods:**

We stimulated Kupffer cells isolated from wild-type (WT) and TLR4^-/-^ mice with recombinant mouse (rm) CIRP (i.e., eCIRP) and assessed supernatant IL-6 and TNFα levels by ELISA. The mRNA expression of iNOS and CD206 for M1 and M2 markers, respectively, was assessed by qPCR. We induced sepsis in WT and CIRP^-/-^ mice by cecal ligation and puncture (CLP) and assessed iNOS and CD206 expression in Kupffer cells by flow cytometry.

**Results:**

eCIRP dose- and time-dependently increased IL-6 and TNFα release from WT Kupffer cells. In TLR4^-/-^ Kupffer cells, their increase after eCIRP stimulation was prevented. eCIRP significantly increased iNOS gene expression, while it did not alter CD206 expression in WT Kupffer cells. In TLR4^-/-^ Kupffer cells, however, iNOS expression was significantly decreased compared with WT Kupffer cells after eCIRP stimulation. iNOS expression in Kupffer cells was significantly increased at 20 h after CLP in WT mice. In contrast, Kupffer cell iNOS expression in CIRP^-/-^ mice was significantly decreased compared with WT mice after CLP. CD206 expression in Kupffer cells was not different across all groups. Kupffer cell M1/M2 ratio was significantly increased in WT septic mice, while it was significantly decreased in CIRP^-/-^ mice compared to WT mice after CLP.

**Conclusion:**

Our data have clearly shown that eCIRP induces Kupffer cell M1 polarization via TLR4 pathway in sepsis, resulting in overproduction of inflammatory cytokines. eCIRP could be a promising therapeutic target to attenuate inflammation by preventing Kupffer cell M1 polarization in sepsis.

## Introduction

Sepsis is defined as life-threatening organ dysfunction caused by dysregulated host immune responses to an infection (1). Sepsis is a major global medical problem as it affects over 50 million people, resulting in approximately 11 million deaths annually worldwide (2). The estimated total healthcare cost of sepsis management in the United States is more than $24 billion per year (3). In sepsis, pathogen-associated molecular patterns (PAMPs) and endogenous damage-associated molecular patterns (DAMPs) are recognized by pattern recognition receptors (PRRs) such as TLR4 and induce aberrant immune responses, resulting in the production of proinflammatory cytokines and chemokines to exacerbate inflammation and organ injury (4, 5). The liver plays an important role in metabolic and immunologic homeostasis and is susceptible to inflammation and injury, and liver dysfunction is recognized to aggravate the severity and mortality of sepsis (6, 7).

Kupffer cells, hepatic resident macrophages, reside in the lumen of the liver sinusoids (8). They are the liver’s first line of defense against bacteria, bacterial endotoxins, and microbial debris from the gastrointestinal tract, acting as a barrier to pathogens entering the liver through the portal vein (8). These cells help maintain tissue homeostasis by phagocytosing bacteria, apoptotic cells, and necrotic cells (9–12). However, they can also contribute to the inflammatory environment by producing significant amount of cytokines and chemokines and recruiting other inflammatory cells such as neutrophils and monocytes (13). Kupffer cells exhibit a range of polarized phenotypes from the proinflammatory M1 phenotype to the anti-inflammatory M2 phenotype under the stimulation of various factors in the microenvironment (14, 15). The inflammatory mediators, including cytokines, chemokines and nitric oxide produced by activated Kupffer cells are known to promote liver injury (7, 13). In sepsis, PAMPs and DAMPs can activate PRRs on Kupffer cells, triggering a dysregulated host response to infection. The aberrant activation of Kupffer cells, as reflected by their over production of proinflammatory mediators, causes significant damage to liver tissues, leading to dysfunction of this vital organ (6, 13). This dysfunction impairs toxin removal, alters metabolism, and dysregulates coagulation, thereby exacerbating sepsis (6, 16).

Cold-inducible RNA-binding protein (CIRP) is an 18-kDa RNA chaperone primarily sequestered in the nucleus. Upon cellular stress such as exposure to endotoxin and hypoxia, CIRP migrates to the cytoplasm and is subsequently released into the extracellular space passively by cell death as well as actively via mechanisms such as the Gasdermin D pore, or exocytosis in the forms of lysosomes and exosomes (17–19). Extracellular CIRP (eCIRP) acts as a DAMP, fueling inflammation and contributing to organ injury (17, 20). eCIRP activates immune cells, such as macrophages, neutrophils, and T cells by binding to TLR4 to promote their proinflammatory responses (17, 20–22). As a result, eCIRP contributes to the pathophysiology of inflammatory disorders (20). It has been shown that eCIRP accounted for liver failure in hepatic ischemia-reperfusion injury as evidenced by increased liver enzymes and histological damage (23, 24). During sepsis, the expression of eCIRP is increased in the liver, resulting in elevated levels of eCIRP in the circulation (17). Inhibition of eCIRP or knockout of CIRP in septic mice improved the outcomes, including, but not limited to, reduced proinflammatory mediators, liver enzymes, and mortality (17, 25). In septic patients, high levels of circulating eCIRP have been shown to correlate with heightened sepsis severity and mortality (17, 26). However, the roles of eCIRP on Kupffer cell activation and polarization remain unknown. Here, we hypothesized that eCIRP promotes proinflammatory M1 polarization of Kupffer cells in sepsis. In this study, we found that eCIRP induced IL-6 and TNFα production and proinflammatory M1 polarization through TLR4. Furthermore, we demonstrated that eCIRP deficiency attenuated Kupffer cell M1 polarization in a mouse model of sepsis. These findings indicate that eCIRP activates Kupffer cells toward a proinflammatory M1 phenotype in sepsis, and targeting eCIRP could be a potential therapeutic approach by regulating inflammatory Kupffer cell polarization.

## Materials and methods

### Mice

C57BL/6 wild-type (WT) mice were purchased from the Charles River Laboratories (Wilmington, MA). Male mice between 8 and 12 weeks of age were used for all experiments. C57BL/6 CIRP^-/-^ mice were originally obtained from Dr. Jun Fujita (Kyoto University, Kyoto, Japan), and C57BL/6 TLR4^-/-^ mice were obtained from Dr. Kevin Tracey (The Feinstein Institutes for Medical Research, Manhasset, NY). Mice were housed in a temperature-controlled room with a 12-hour light cycle and provided with standard laboratory chow and drinking water. All experimental procedures followed the guidelines for the use of laboratory animals set by the National Institutes of Health. Our Institutional Animal Care and Use Committee approved all experiments of this study.

### Isolation of Kupffer cells

Kupffer cells were isolated from mouse liver using collagenase digestion method previously described with minor modifications (27, 28). After laparotomy, the portal vein was cannulated, and the liver was perfused with HBSS containing 0.5 mM EGTA prewarmed at 37°C, followed by perfusion with prewarmed HBSS-CaCl_2_ (1mM) containing 0.5 mg/mL collagenase type 4 (Worthington Biochemical, Lakewood, NJ). Perfused liver tissue was gently dispersed in a 100 mm cell culture plate using a pair of ophthalmic forceps, and the cell suspension was filtered through a 70 μm cell strainer. The filtrate was subjected to differential centrifugation to separate non-parenchymal cells (NPCs) from hepatocytes. After centrifugation of the cell suspension at 50 × g for 2 min at 4°C twice, the supernatant was centrifuged at 450 × g for 5 min at 4°C to obtain NPCs. The NPCs were suspended in 25% Percoll (Cytiva, Marlborough, MA), layered onto 50% Percoll, and centrifuged at 850 × g for 20 min at 15°C. The interface containing Kupffer cells was collected and further purified by selective adherence to cell culture plates for 3 h in RPMI 1640 medium with 10% heat-inactivated FBS, 2 mM glutamine, and 1% penicillin-streptomycin.

### Enzyme-linked immunosorbent assay

IL-6 and TNFα were evaluated in Kupffer cell culture supernatants using mouse-specific enzyme-linked immunosorbent assay (ELISA) kits (BD Biosciences, San Jose, CA) according to the manufacturer’s instructions.

### Real-time quantitative reverse transcription polymerase chain reaction

Total RNA was extracted from Kupffer cells using an Illustra RNAspin mini kit (GE Healthcare, Chicago, IL) according to the manufacturer’s instructions. Reverse transcription was performed using an iScript cDNA Synthesis Kit (Bio-rad, Hercules, CA). Polymerase chain reaction (PCR) reaction was performed in a final volume of 20 μL containing 0.06 μM of forward and reverse primer, 2 μg of cDNA, nuclease-free water, and SYBR Green PCR Master Mix (Applied Biosystems, Thermo Fisher Scientific). Amplification and analysis were conducted in a Step One Plus real-time PCR machine (Applied Biosystems, Thermo Fisher Scientific). Mouse β-actin mRNA served as the internal control for amplification. Relative gene expression was calculated using 2^-ΔΔCT^ method. Relative expression of mRNA was determined as the fold change in comparison to the WT-PBS control. The primer sequences for this study were as follows: iNOS (NM_007705), 5’-GGCAAACCCAAGGTCTACGTT-3’ (forward), and 5’-GAGCACGCTGAGTACCTCATTG-3’ (reverse); β-actin (NM_007393), 5’-CGTGAAAAGATGACCCAGATCA-3’ (forward), and 5’-TGGTACGACCAGAGGCATACAG-3’ (reverse); CD206 (NM_008625.2), 5’-CCCAAGGGCTCTTCTAAAGCA-3’ (forward), and 5’-CGCCGGCACCTATCACA-3’ (reverse).

### Murine model of polymicrobial sepsis

Polymicrobial sepsis was induced in mice by cecal ligation and puncture (CLP) as previously described (17, 29). Briefly, mice were anesthetized with isoflurane inhalation, and a midline abdominal incision was made. The cecum was exposed and ligated 1 cm proximal to the end with a 4-0 silk suture. The ligated distal part of the cecum was punctured twice with a 22-gauge needle, and a small amount of fecal material was extruded from the punctures. The cecum was returned to the abdominal cavity, and the abdomen was closed in two layers. After surgery, the mice received a subcutaneous injection of 0.5 mL of normal saline and 0.05 mg/kg body weight of buprenorphine and were then returned to their cages with access to food and drinking water.

### Flow cytometry

To evaluate iNOS and CD206 expression in Kupffer cells isolated from an *in vivo* sepsis model, mouse liver NPCs were isolated and analyzed by flow cytometry. The mouse liver tissues were enzymatically and mechanically digested using the Liver Dissociation Kit (Cat. No.: 130105807; Miltenyi Biotec, Bergisch Gladbach, Germany) in a gentleMACS Octo Tissue Dissociator (Miltenyi Biotec) for about 30 min according to the manufacturer’s protocol. The dissociated cell suspensions were filtered through a 70 μm strainer. After centrifugation of the cell suspension at 50 × g at 4°C twice, the supernatant was centrifuged at 450 × g for 5 min at 4°C to obtain NPCs. Red blood cells were removed using the red blood cell lysis buffer (Cat. No.: 555899; BD Biosciences). Isolated NPCs were stained with PerCP/Cy5.5 anti-mouse CD45 Ab (clone: I3/2.3; BioLegend), FITC anti-mouse/human CD11b Ab (clone: M1/70; BioLegend), APC anti-mouse F4/80 Ab (clone: BM8; BioLegend), and BV421 anti-mouse CD206 Ab (clone: C068C2; BioLegend) and then fixed in Fluorofix buffer (Cat. No.: 422101; BioLegend), followed by the intracellular staining with PE anti-mouse iNOS Ab (Cat. No.: sc-7271; Santa Cruz Biotechnology, Dallas, TX) in Permeabilization Wash Buffer (Cat. No.: 421002; BioLegend). Kupffer cells were identified as the F4/80^hi^ and CD11b^lo^ population within CD45^+^ cells. iNOS and CD206 expression in Kupffer cells was determined by flow cytometry. Flow cytometry data were acquired using a BD LSRFortessa flow cytometer (BD Biosciences) and analyzed with Flowjo software (Tree Star, Ashland, OR).

### Statistical analysis

Data analysis was performed using GraphPad Prism graphing and statistical software (GraphPad Software, LLC, San Diego, CA). The presented data in the figures are expressed as mean ± SEM and were compared using one- or two-way analysis of variance (ANOVA) followed by Tukey’s multiple comparison test for multiple groups. A p-value of < 0.05 was considered statistically significant for comparisons between experimental groups.

## Results

### eCIRP activates Kupffer cells to induce IL-6 and TNFα production

**Figure 1:**
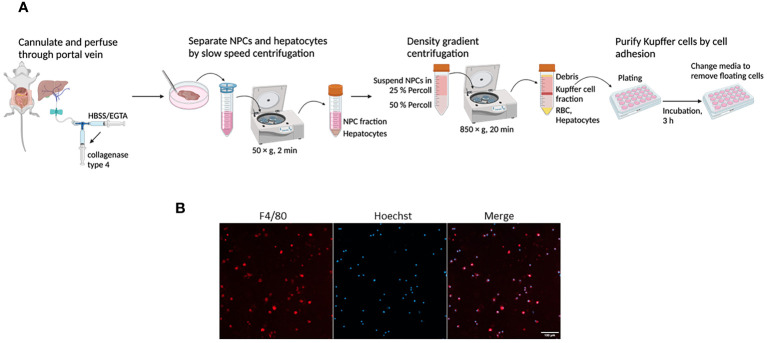
Purification and identification of isolated murine Kupffer cells. **(A)** Schematic overview of Kupffer cell isolation. The portal vein was cannulated to perfuse the liver, followed by *in situ* collagenase digestion. After liver digestion, NPCs were separated from the hepatocytes by differential centrifugation. Kupffer cells were purified from the NPCs by Percoll gradient centrifugation and further selective adherence to the culture plate. Kupffer cells were isolated from WT mice and cultured for 1 day, then the cells were stained with F4/80 Ab. The nuclei were stained with Hoechst 3334. **(B)** Representative confocal microscopy images showing the fluorescence of F4/80 (red) and Hoechst 3334 (blue). Scale bar, 100 μm.

**Figure 2:**
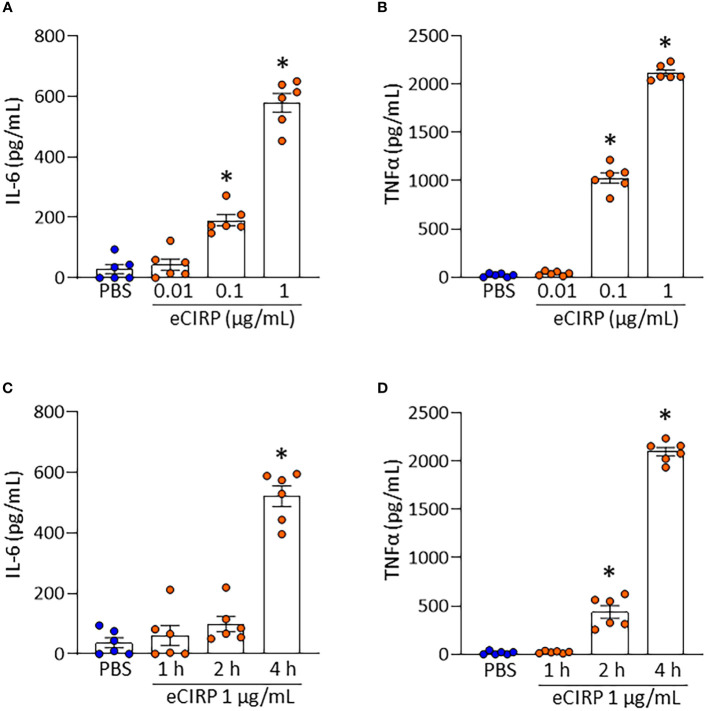
eCIRP increases IL-6 and TNFα production in Kupffer cells. Kupffer cells isolated from WT mice were stimulated with **(A, B)** PBS or eCIRP (0.01, 0.1 or 1 μg/mL) for 4 h and **(C, D)** PBS or 1 μg/mL of eCIRP for 1, 2 or 4 h. After stimulation, the cell culture supernatants were collected. The levels of **(A, C)** IL-6 and **(B, D)** TNFα in the supernatants were evaluated by ELISA. Experiments were repeated two times and all the data obtained were used for analysis. Data are expressed as mean ± SEM (n=6 samples/group). The groups were compared by one-way ANOVA and Tukey’s multiple comparison test (*p < 0.05 *vs*. PBS).

We have isolated Kupffer cells from normal mouse liver and confirmed the identity by immunofluorescence (**Figures 1A, B**). To investigate the effect of eCIRP on Kupffer cells, we stimulated Kupffer cells with different concentrations of eCIRP and collected the supernatants at different time points to assess proinflammatory cytokines IL-6 and TNFα. After 4 h of eCIRP stimulation, Kupffer cells showed a significant increase in IL-6 and TNFα production at 0.1 μg/mL of eCIRP stimulation and a further increase at 1 μg/mL of eCIRP treatment (**Figures 2A, B**). Moreover, we observed that eCIRP-stimulated Kupffer cells exhibited a time-dependent increase in IL-6 and TNFα production (**Figures 2C, D**). Taken together, eCIRP activates Kupffer cells to release proinflammatory cytokines in a time- and dose-dependent manner.

### eCIRP induces IL-6 and TNFα production in Kupffer cells via TLR4

**Figure 3:**
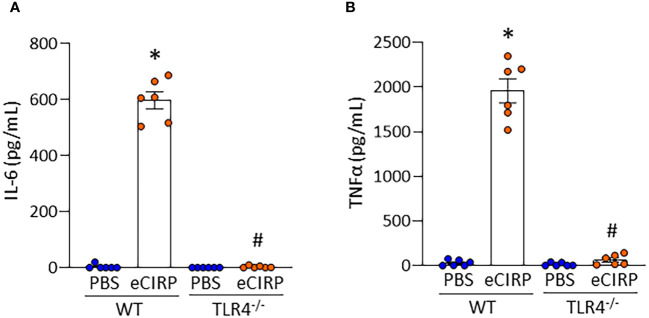
eCIRP induces IL-6 and TNFα production in Kupffer cells through TLR4. Kupffer cells isolated from WT and TLR4^-/-^ mice were stimulated with PBS or 1 μg/mL of eCIRP for 4 h. After stimulation, cell culture supernatants were collected. The levels of **(A)** IL-6 and **(B)** TNFα in the supernatants were assessed by ELISA. Experiments were repeated two times and all the data obtained were used for analysis. Data are expressed as mean ± SEM (n=6 samples/group). The groups were compared by two-way ANOVA and Tukey’s multiple comparison test (*p < 0.05 *vs*. WT PBS, ^#^p < 0.05 *vs*. WT eCIRP).

We have previously shown that eCIRP induces macrophages to release proinflammatory cytokines by stimulating TLR4 (17). Therefore, we investigated the involvement of TLR4 in eCIRP-induced proinflammatory cytokine production of Kupffer cells. We stimulated Kupffer cells isolated from WT and TLR4^-/-^ mice with eCIRP. After 4 h of stimulation with eCIRP, we assessed IL-6 and TNFα levels in the supernatant of Kupffer cell culture. eCIRP stimulation of WT Kupffer cells resulted in a significant increase in the release of IL-6 and TNFα (**Figures 3A, B**). However, we found a significant decrease in the IL-6 and TNFα release in TLR4^-/-^ Kupffer cells compared with WT Kupffer cells after eCIRP stimulation (**Figures 3A, B**). Thus, our data reveal that eCIRP induces proinflammatory cytokine release from Kupffer cells in a TLR4-dependent manner.

### eCIRP induces Kupffer cell polarization toward M1 phenotype through TLR4 pathway

**Figure 4:**
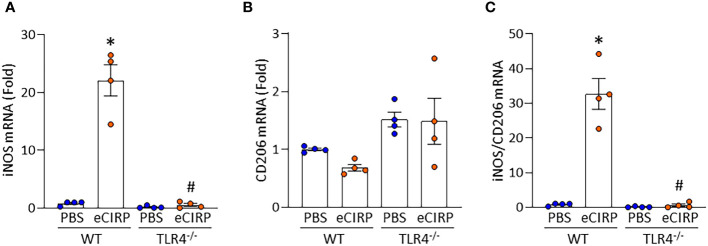
eCIRP promotes M1 polarization of Kupffer cells via TLR4. Kupffer cells isolated from WT and TLR4^-/-^ mice were stimulated with PBS or 1 μg/mL of eCIRP for 4 h. **(A)** iNOS and **(B)** CD206 mRNA expression was assessed by qPCR and normalized to β-actin expression. Results are represented as fold induction compared with the normalized values of WT PBS-treated cells. **(C)** The ratio of iNOS to CD206 mRNA expression was evaluated. Experiments were repeated two times and all the data obtained were used for analysis. Data are expressed as mean ± SEM (n=4 samples/group). The groups were compared by two-way ANOVA and Tukey’s multiple comparison test (*p < 0.05 *vs*. WT PBS, ^#^p < 0.05 *vs*. WT eCIRP).

We next aimed to investigate whether eCIRP induces Kupffer cell polarization toward a proinflammatory M1 phenotype. We assessed iNOS as an M1 marker and CD206 as an M2 marker in Kupffer cells isolated from WT and TLR4^-/-^ mice after 4 h of eCIRP stimulation. We found that the mRNA expression of iNOS was significantly increased in WT Kupffer cells after eCIRP stimulation compared with PBS-treated cells. In contrast, TLR4^-/-^ Kupffer cells stimulated with eCIRP did not exhibit an increase in iNOS expression compared with PBS-treated controls. When comparing the eCIRP-stimulated groups, iNOS expression was significantly lower in TLR4^-/-^ Kupffer cells than WT Kupffer cells (**Figure 4A**). However, CD206 mRNA expression was not significantly different between PBS- and eCIRP-treated groups in both WT and TLR4^-/-^ Kupffer cells (**Figure 4B**). To further evaluate the polarization of eCIRP-stimulated Kupffer cells, we examined the iNOS/CD206 ratio (M1/M2). After eCIRP stimulation, iNOS/CD206 ratio was significantly increased in WT Kupffer cells but significant decreased in TLR4^-/-^ Kupffer cells compared with WT Kupffer cells (**Figure 4C**). These findings suggest that eCIRP polarizes Kupffer cells toward the M1 proinflammatory phenotype through TLR4.

### eCIRP deficiency alleviates sepsis-induced M1 polarization of Kupffer cells

**Figure 5:**
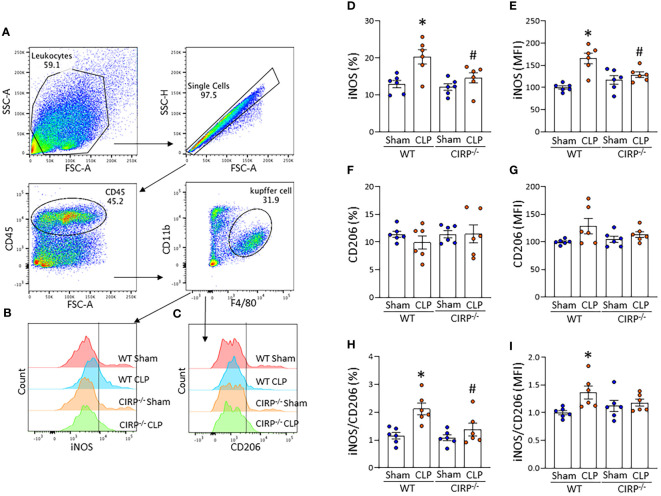
eCIRP deficiency results in decreased sepsis-induced M1 polarization of Kupffer cells. WT and CIRP^-/-^ mice underwent CLP or sham operation. After 20 h, liver samples were collected to assess iNOS and CD206 expression of Kupffer cells using flow cytometry. Representative **(A)** dot blots of the gating strategy and **(B, C)** histograms of **(B)** iNOS and **(C)** CD206 expression are shown. The bar diagram showing **(D, F)** frequencies and **(E, G)** mean immunofluorescence intensity (MFI) of **(D, E)** iNOS and **(F, G)** CD206 expression. The ratio of **(H)** frequency of iNOS to CD206 expressing cells and the ratio of **(I)** MFI of iNOS to CD206 expression are shown. All experiments were performed three times, and all data were used for analysis. Data are expressed as mean ± SEM (n=6 mice/group). The groups were compared by two-way ANOVA and SNK Tukey’s multiple comparison test (*p < 0.05 *vs*. WT sham, ^#^p < 0.05 *vs*. WT CLP mice).

We then sought to determine the role of eCIRP on Kupffer cell polarization in sepsis using a CLP model. Sepsis was induced in WT and CIRP^-/-^ mice by CLP, and the liver was harvested 20 h after CLP operation. We isolated NPCs from the liver and assessed the expression of iNOS and CD206 in Kupffer cells by flow cytometry (**Figures 5A–C**). The frequency of iNOS^+^ cells and mean immunofluorescence intensity (MFI) of iNOS expression in Kupffer cells were significantly increased after sepsis in WT mice (**Figures 5D, E**). Interestingly, the iNOS expression in Kupffer cells of CIRP^-/-^ mice did not show significant difference after sepsis compared with CIRP^-/-^ sham mice. When we compared iNOS expression between the septic mice, we found significantly decreased iNOS expression in Kupffer cells of CIRP^-/-^ septic mice compared to WT mice with sepsis (**Figures 5D, E**). Meanwhile, CD206 expression on Kupffer cells was not significantly different across all groups (**Figures 5F, G**). iNOS (%)/CD206 (%) ratio in Kupffer cells was significantly increased in WT septic mice compared with WT sham mice but significantly decreased in CIRP^-/-^ septic mice compared with WT septic mice (**Figure 5H**). iNOS (MFI)/CD206 (MFI) ratio was significantly increased after sepsis in WT mice but was not significantly different after sepsis in CIRP^-/-^ mice (**Figure 5I**). These data indicate that eCIRP plays a crucial role in the induction of Kupffer cell polarization toward a proinflammatory M1 phenotype in sepsis.

## Discussion

**Figure 6:**
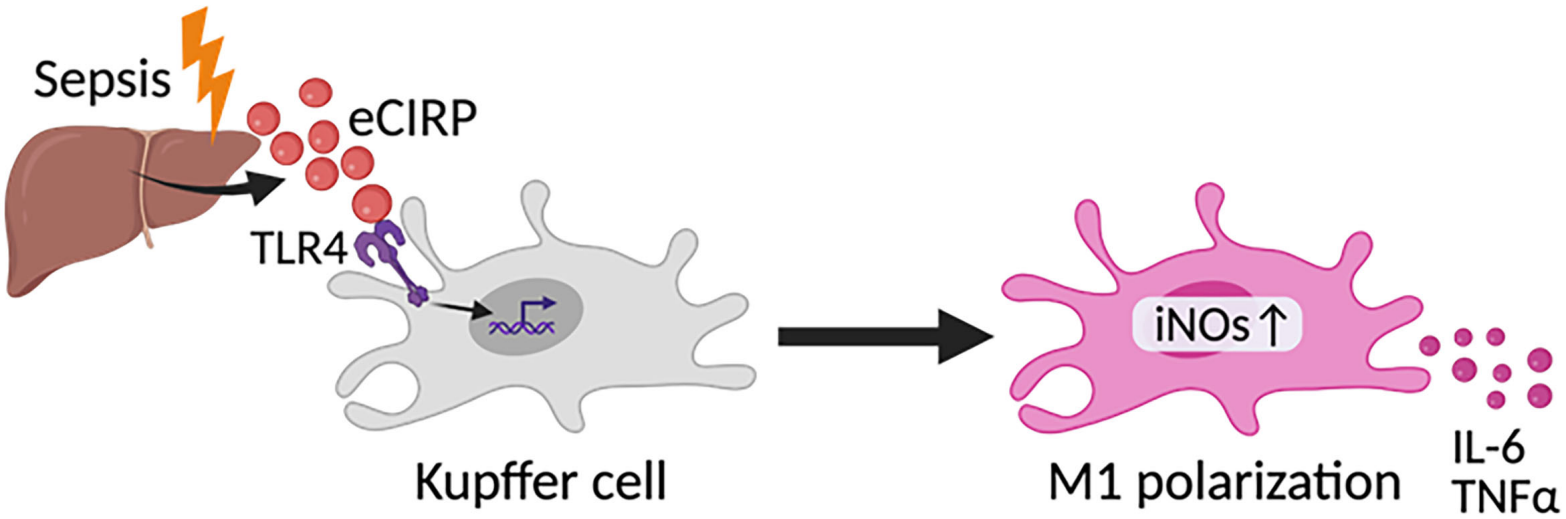
Summary of findings. In sepsis, eCIRP activates TLR4 on Kupffer cells to promote M1 polarization as reflected by increased iNOS, leading to the production of proinflammatory cytokines IL-6 and TNFα.

In the current study, we have demonstrated that eCIRP polarizes Kupffer cells to the M1 phenotype, promoting the production of the proinflammatory cytokines IL-6 and TNFα. Furthermore, our findings indicate that eCIRP plays a critical role in polarizing Kupffer cells to the M1 phenotype during sepsis (**Figure 6**). Kupffer cells, the resident macrophages of the liver, play an important role in the innate immune response (8). These cells undergo distinct phenotypic polarizations, M1 and M2, in response to various endogenous and exogenous stimuli under different disease conditions (14, 15). M1 type of Kupffer cells, which express iNOS, promote inflammation by producing proinflammatory cytokines such as TNFα and IL-6, while M2 Kupffer cells, which express CD206, exhibit anti-inflammatory properties by producing IL-10 and TGF-β (9, 30–32). In this study, we demonstrated polarization of Kupffer cells toward the M1 phenotype in sepsis as evidenced by increased iNOS expression and iNOS/CD206 ratio. Furthermore, Kupffer cells from CIRP^-/-^ mice showed decreased iNOS expression and iNOS/CD206 ratio compared with WT mice after CLP, confirming the impact of eCIRP on Kupffer cell M1 polarization in sepsis. M1 Kupffer cells have the potential to produce significant amounts of proinflammatory cytokines, including IL-6 and TNFα (31). These soluble mediators dysregulate the host immune response by inducing aberrant activities and immunogenic cell death in various cell types not only in the surrounding environment but also in the circulation and remote organs (33).

Our previous studies have shown that eCIRP levels in the liver and circulation are elevated in septic mice (17). We have also previously demonstrated that eCIRP accounts for increased cytokine and chemokine levels, neutrophil influx, and tissue injury in the liver of a hepatic ischemia-reperfusion injury model (23, 24). Here, we showed that eCIRP-stimulated Kupffer cells produce proinflammatory cytokines and eCIRP induces M1 polarization of Kupffer cells in septic mice. Collectively, it is strongly suggested that increased eCIRP in the liver activates Kupffer cells and polarizes them into the M1 phenotype releasing proinflammatory cytokines to aggravate liver injury in sepsis. eCIRP has been shown to bind to TLR4 and activate macrophages to release cytokines and chemokines (17). Although eCIRP also stimulates other PRRs which contribute to the inflammation in sepsis, such as triggering receptor expressed on myeloid cells-1 (TREM-1) (34), the present study showed TLR4^-/-^ Kupffer cells were barely responsive to eCIRP, indicating the predominant role of the TLR4-mediated pathway in the polarization of Kupffer cells toward the M1 phenotype. The complete inhibition of cytokine upregulation and polarization markers in TLR4 knockout Kupffer cells may stem from the abundance of TLR4 in these cells compared to other receptors where eCIRP can bind. Nevertheless, inhibiting the eCIRP-TLR4 axis could interfere with the TREM-1 pathway since TREM-1 can act as an amplifier of TLR4 signal transduction and synergistically enhances inflammation (35, 36). Consequently, we anticipate that Kupffer cells isolated from TREM-1 knockout mice would exhibit similar data to TLR4 knockout cells, as both receptors likely mediate optimal induction when working in concert. In peritoneal macrophages and other innate immune cells such as neutrophils, we examined the TLR4 and TREM-1 pathways to elucidate eCIRP’s role in promoting a pro-inflammatory phenotype (17, 21, 34, 37). Our findings regarding Kupffer cell polarization could potentially be correlated with pathways previously identified as contributing to the inflammatory phenotype in innate immune cells.

Since eCIRP plays a significant role in the pro-inflammatory polarization of Kupffer cells in sepsis, targeting eCIRP by inhibitors may have the potential to mitigate liver injury. We have previously generated synthetic peptides and oligonucleotides which inhibit eCIRP (17). C23 is a 15-aa peptide derived from eCIRP that competitively inhibit the binding of eCIRP to TLR4. It has been shown that C23 significantly attenuated cytokine production from eCIRP-stimulated macrophages (38). Dodeca-adenisine (A_12_), an engineered poly(A) mRNA mimic, selectively and strongly interacts with the RNA-binding motif of eCIRP to neutralize eCIRP (31). A_12_ prevents eCIRP from binding to TLR4, thereby attenuating eCIRP-induced inflammatory cytokine production in macrophages (25). These inhibitors could also inhibit proinflammatory cytokine production and polarization of eCIRP-challenged Kupffer cells in the similar manner. Furthermore, exploring the therapeutic effects of the eCIRP inhibitors on Kupffer cell activation and proinflammatory polarization in sepsis-induced liver injury and their mechanisms of action would be of interest in the future.

In this study, we did not evaluate the M1 polarization of Kupffer cells at time points longer than 4 h in *in vitro* experiments and 20 h in *in vivo* experiments. During sepsis, M1 macrophages can exhibit compensation for inflammation by acquiring an M2-like immunosuppressive phenotype that increases the risk of secondary infection and subacute or late mortality (39, 40). LPS polarizes macrophages to the M1 phenotype at the early phase, whereas M2 macrophages are generated after prolonged or repeated exposure to LPS, resulting in endotoxin tolerance (39, 40). Our previous study showed that eCIRP-challenged macrophages polarized to the M2 phenotype after 24 to 48 h, thereby inducing endotoxin tolerance (41). The same study also demonstrated that mice exhibited endotoxin tolerance 72 h post CLP. In this study, we focused on the state of Kupffer cells at 4 and 20 h after *in vitro* eCIRP stimulation and *in vivo* sepsis, respectively, to elucidate the role of eCIRP in M1 inflammatory polarization. Future studies at longer time points may reveal the impact of eCIRP on the plasticity of Kupffer cells in sepsis. In our current study, we demonstrated Kupffer cell activation by eCIRP in an infectious model of polymicrobial sepsis. It is important to note that sepsis can also arise from monobacterial insults as well as sterile tissue injuries such as gut ischemia/reperfusion injury. Further investigations into eCIRP’s influence on Kupffer cell polarization within these preclinical models could offer valuable insights into the underlying mechanisms of these disease conditions.

When determining the frequency of M1- and M2-associated markers in Kupffer cells, we evaluated iNOS and CD206 expressions separately, the same method as a previous study evaluating M1/M2 polarization of Kupffer cells using these markers (42). In this way, iNOS and CD206 double-positive cells were counted in both phenotypes, potentially causing bias in the analysis. Nevertheless, quadrant plots clearly showed that Kupffer cells skewed toward the M1 phenotype specifically in WT CLP mice (data not shown). iNOS^+^CD206^+^ Kupffer cells could be considered as transient or hybrid population. The characteristics and roles of this subset would be worth investigating in the future studies. It should also be noted that large population of Kupffer cells did not express M1 or M2 marker even in CLP mice, indicating they remained unpolarized, i.e., M0. This could be due to the time point of our study. At the acute phase of sepsis, the phenotype of Kupffer cells is primarily affected by the innate immune system. On the contrary, higher percentage of polarized Kupffer cells could be observed at the later phase, where the adaptive immunity also plays a critical role.

In conclusion, eCIRP induces M1 polarization of Kupffer cells during sepsis, leading to the production of inflammatory cytokines. Therefore, eCIRP may present a promising therapeutic target for mitigating sepsis-induced inflammatory responses and liver injury by preventing Kupffer cell M1 polarization.

## Data availability statement

The original contributions presented in the study are included in the article. Further inquiries can be directed to the corresponding author.

## Ethics statement

The animal study was approved by Institutional Animal Care and Use Committee of the Feinstein Institutes for Medical Research. The study was conducted in accordance with the local legislation and institutional requirements.

## Author contributions

JS: Conceptualization, Data curation, Investigation, Methodology, Project administration, Visualization, Writing – original draft. AM: Conceptualization, Data curation, Investigation, Methodology, Project administration, Visualization, Writing – original draft. YL: Investigation, Methodology, Visualization, Writing – review & editing. MA: Conceptualization, Funding acquisition, Methodology, Project administration, Resources, Supervision, Validation, Writing – original draft, Writing – review & editing. PW: Funding acquisition, Project administration, Resources, Supervision, Validation, Writing – review & editing.
